# A 120,000-year long climate record from a NW-Greenland deep ice core at ultra-high resolution

**DOI:** 10.1038/s41597-021-00916-9

**Published:** 2021-05-26

**Authors:** Vasileios Gkinis, Bo M. Vinther, Trevor J. Popp, Thea Quistgaard, Anne-Katrine Faber, Christian T. Holme, Camilla-Marie Jensen, Mika Lanzky, Anine-Maria Lütt, Vasileios Mandrakis, Niels-Ole Ørum, Anna-Sofie Pedersen, Nikol Vaxevani, Yongbiao Weng, Emilie Capron, Dorthe Dahl-Jensen, Maria Hörhold, Tyler R. Jones, Jean Jouzel, Amaëlle Landais, Valérie Masson-Delmotte, Hans Oerter, Sune O. Rasmussen, Hans Christian Steen-Larsen, Jørgen-Peder Steffensen, Árný-Erla Sveinbjörnsdóttir, Anders Svensson, Bruce Vaughn, James W. C. White

**Affiliations:** 1 Physics of Ice Climate and Earth, Niels Bohr Institute, University of Copenhagen, Tagensvej 16, 2200 Copenhagen, Denmark; 2 Geophysical Institute and Bjerknes Centre for Climate Research, University of Bergen, Allégaten 70, 5007 Bergen, Norway; 3 Climate and Environmental Physics, Physics Institute, Sidlerdtrasse 5, 3012 Bern, Switzerland; 4 Department of Geosciences, University of Oslo, Sem Sæ lands Vei 1, 0371 Oslo, Norway; 5 Institut des Géosciences de l’Environnement, Université Grenoble Alpes, CNRS, IRD, G-INP, 38000 Grenoble, France; 6 Centre for Earth Observation Science, 535 Wallace Building, University of Manitoba, Winnipeg, MB R3T 2N2 Canada; 7 Alfred-Wegener-Institut Helmholtz-Zentrum für Polar- und Meeresforschung, Am Handelshafen 12, 27570 Bremerhaven, Germany; 8 Institute of Arctic and Alpine Research, University of Colorado, Boulder, Colorado, 80309-0450 USA; 9 Laboratoire des Sciences du Climat et de l’Environnement (LSCE), Institut Pierre Simon Laplace (CEA-CNRS-UVSQ UMR 8212), Université Paris Saclay, Gif-sur-Yvette, France; 10 Institute of Earth Sciences, University of Iceland, Sturlugata 7, Reykjavik, Iceland

**Keywords:** Cryospheric science, Palaeoclimate, Geochemistry

## Abstract

We report high resolution measurements of the stable isotope ratios of ancient ice (*δ*^18^O, *δ*D) from the **N**orth Greenland **Eem**ian deep ice core (NEEM, 77.45° N, 51.06° E). The record covers the period 8–130 ky b2k (y before 2000) with a temporal resolution of ≈0.5 and 7 y at the top and the bottom of the core respectively and contains important climate events such as the 8.2 ky event, the last glacial termination and a series of glacial stadials and interstadials. At its bottom part the record contains ice from the Eemian interglacial. Isotope ratios are calibrated on the SMOW/SLAP scale and reported on the GICC05 (Greenland Ice Core Chronology 2005) and AICC2012 (Antarctic Ice Core Chronology 2012) time scales interpolated accordingly. We also provide estimates for measurement precision and accuracy for both *δ*^18^O and *δ*D.

## Abstract (summary)

**Table:** 

Measurement(s)	isotope analysis • water ice core
Technology Type(s)	cavity ring-down spectroscopy
Factor Type(s)	δ18O • δD
Sample Characteristic - Location	Greenland Ice Sheet

Machine-accessible metadata file describing the reported data: 10.6084/m9.figshare.14216441

## Background & Summary

The isotopic composition of ice from deep cores drilled on Greenland and Antarctica has traditionally been used as a proxy for past temperatures^1–4^ offering a picture of past climate that extends as far as 120,000 y in the past^5^ for the case of Greenland and 800,000 y for the case of Antarctica^6^. Typically expressed with the *δ* notation (isotopic abundances are reported as deviations of a sample’s isotopic ratio relative to that of a reference water (e.g. VSMOW) expressed in per mille (‰) as: *δ*^*i*^ = $\left(\frac{{}^{i}{R}_{{\rm{s}}{\rm{a}}{\rm{m}}{\rm{p}}{\rm{l}}{\rm{e}}}}{{}^{i}{R}_{{\rm{V}}{\rm{S}}{\rm{M}}{\rm{O}}{\rm{W}}}}-1\right)\times 1000$  where ${}^{2}R=\frac{{}^{2}{\rm{H}}}{{}^{1}{\rm{H}}}$  and ${}^{18}R=\frac{{}^{18}{\rm{O}}}{{}^{16}{\rm{O}}})$, the isotopic composition records hydrological cycle changes spanning centennial to millennial scales. At sufficiently high resolution, water isotope records can be used in order to resolve climatic signals at higher frequencies, investigate abrupt climate events, count annual layers for the purpose of ice core chronologies and extract paleoclimate information stored in their spectral signature^7–14^.

Stable water isotope analysis has traditionally been performed with Isotope Ratio Mass Spectrometry (IRMS) utilising an array of different implementations, each one presenting advantages and disadvantages with respect to precision, accuracy, sample consumption and throughput, as well as ease of use and labour intensity^15–19^. With the advent of fast electronics and room-temperature, turn-key, solid state laser sources, there emerged various optical spectroscopic techniques, typically in the near Infra-Red spectral region that allowed for quasi-simultaneous analysis of the three isotope ratios ^2^H/^1^H, ^17^O/^16^O and ^18^O/^16^O on the same water sample^20,21^. Commercial instruments using Cavity Enhanced Spectroscopic techniques allowed for routine simultaneous measurements of *δ*^18^O and *δ*D using a greatly reduced amount of sample and with precision and accuracy comparable, if not better than that of IRMS^22,23^. The new techniques offered the possibility for *in-situ* measurements of the isotopic composition of vapour^24–26^ while in combination with continuous melter systems, and micro-volume flash vaporisers, they yielded ice core data sets of unprecedented resolution^23,27–31^.

Here, we present a high-resolution (0.05 m) water isotope (*δ*^18^O and *δ*D) record^32^ from the Greenland NEEM ice core, drilled from 2007 to 2012 at 77.45°N–51.06°W. The record covers the segment of the ice core below 1210.5 m - a depth that corresponds to an age of 8000 y b2k (y before 2000 AD)- and extends to a final depth of 2536.5 m. The oldest age of the record is 129,258 y b2k at the depth of 2432.15 m. We report ages on the GICC05 (ages 8–121.446 ky b2k) and the AICC2012 (ages 60–129.258 ky b2k) time scales interpolated accordingly. Beyond that point and for the deepest 104 m, an age scale cannot be reconstructed as the record is too disturbed due to ice folds^33^.

Isotopic analysis has been performed using Laser Spectroscopy and in particular three different versions of the Cavity Ring Down Spectrometers (CRDS) from Picarro Inc. (L2120-i, L2130-i, L2140-i). All measurements are reported on the international VSMOW-SLAP (VSMOW and SLAP refer to the International Atomic Energy reference water materials and stand for Vienna Mean Ocean Water and Standard Light Antarctic Precipitation) isotope scale after careful calibration using a triplet of local standards calibrated against the primary International Atomic Energy Agency reference materials. We describe the methodology of the measurement including information on instrument calibration procedures and an accuracy and precision assessment.

The dataset will be useful for future studies of past climate variability on long (millennial) and short (decadal to annual) time scales. Studies focusing on the spectral properties of the isotopic signal will also benefit from the high resolution apparent in the record. The record includes significant past-climate events such as the 8.2 ky climatic transition, the last Glacial Termination, as well as the series of Glacial Stadial-Interstadials of the last 120 ky (GI-1, GS-1 to GI-25c, GS-26)0^34^.

## Methods

### Discrete Ice Core Sampling

**Fig. 1:**
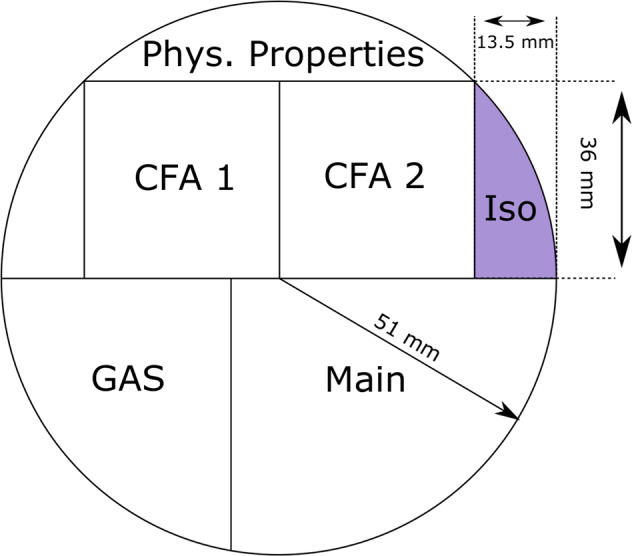
Sampling scheme for the NEEM ice core. CFA refers to the section sampled for Continuous Flow Analysis and Iso refers to the section sampled for water isotope analysis.

The sampling of the ice core for water isotope analysis was performed in the field during the field seasons 2009–2012. A wedge with a cross section of approximately 10 cm^2^ (roughly 12% of the cross section of the ice core) was cut using a band saw with a resolution of 0.025 m for the top 601.7 m and 0.05 m for the interval 601.75–2536.5 (Fig. 1). The dataset presented here, has a resolution of 0.05 m. The ice samples were stored and transported to Copenhagen, Denmark frozen in plastic bags for further preparation and isotopic analysis.

Subsequently, and after arrival to the Stable Isotope Lab at the Niels Bohr Institute, the samples were melted at room temperature in air-tight containers and transferred to 10 ml PE-LD narrow mouth bottles (**VITLAB-138093**). From that point, the samples were stored frozen until the time of isotopic analysis using CRDS. Prior to analysis, 200 *μ*l of sample were transferred from the PE-LD bottles to 2 ml short thread glass vials with integrated 300 *μ*l micro-inserts (**La-Pha-Pack 11092357**) sealed with triple layer Teflon/Silicon/Teflon caps (**La-Pha-Pack 09150480**).

### Stable isotope analysis

#### Cavity ring down spectroscopy

Isotope analysis is performed with Cavity Ring Down Spectroscopy (**CRDS**) in the near Infra-Red region. For this study, we have used three different models of the Picarro L21XX-i analyser namely the L2120-i, L2130-i and L2140-i. The record contains a total of 26,519 data points acquired with the three instruments. The exact number of data points per instrument is given in Table 1.

**Table 1:** Types of instruments used and their relative contribution.

Instrument	Points measured	Coverage [%]
L2120-i	6857	25.9
L2130-i	14384	54.2
L2140-i	5027	19
Interpolated	251	0.9
Total	26519	100.0

All three models follow the same spectroscopic principle for the measurement of isotope ratios and are very similar with respect to their performance. As we show in section 4, the L2130-i and L2140-i models reach higher levels of precision and accuracy when compared to the older generation L2120-i instrument. CRDS measurements are performed in the vapour phase with the sample continuously flowing through a high finesse optical cavity. The cavity consists of three high reflectivity mirrors in a V-shape configuration^22^. A continuous emission diode laser is coupled to the optical cavity in which the temperature and the pressure are accurately controlled (75 Torr and 85 °C). The light leaking out of one of the cavity mirrors is measured by a solid state photodiode. When the light intensity reaches a certain threshold, the laser is abruptly switched off and the light intensity signal on the photodiode decays exponentially. The time constant of the exponential decay, commonly referred to as ‘ring-down time’ depends on the concentration of the absorbant molecules present in the optical cavity. For this study we have performed measurements of *δ*^18^O and *δ*D, thus the CRDS protocol used, takes into account absorption lines of the ^1^H_2_^16^O, ^1^H_2_^18^O and ^1^H^2^H^16^O isotopologues.

#### Sample vaporisation and injection method

Vaporisation of the sample is performed using a high throughput, low volume vaporiser (Picarro-A0212 – discontinued model as of 2016). The A0212 vaporiser utilises a continuous vaporisation approach similar to that developed for the Continuous Flow *δ*^18^O / *δ*D analysis^23^. A nano-flow of the liquid sample generated by a micro-volume syringe, is vaporised continuously on a hot stainless steel plate at a temperature of 170 °C within a stream of dry air ([H_2_O] < 30 ppm). The CRDS analyser pumps the water vapour sample into the optical cavity with a flow rate of 30 cc/min (STP) via an open split connection with the inlet of the vaporiser.

**Fig. 2:**
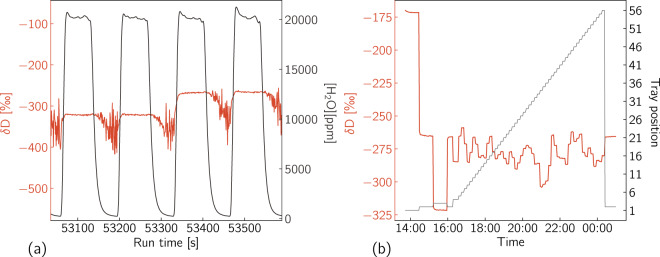
Vaporiser pulse example and run tray configuration. (**a**) A sequence of four vaporiser pulses focused at the transition from the “−40” to the “NEEM” standard water. (**b**) Vial tray configuration and raw *δ*D data of a full measurement run as a function of time.

A total of 3.8 *μ*l of liquid water sample is injected with a liquid flow of 50 nl/s using a 10 *μ*l syringe (**SGE 10R-C/F5**). The stability of the sample injection is a critical prerequisite for achieving high quality measurements with the continuous vaporisation method. An injection cleaning procedure is performed on a daily basis using an ultrasonic acetic acid bath followed by several manual rinses with deionised water. The thorough daily cleaning is vital for achieving measurements of high quality. The injection is performed using a CTC GC-PAL autosampler (PAL hereafter) with an injection method that results in a pulse duration of ≈80 sec and a pulse amplitude of 20,000 ppm. The amount of time required for one injection, including the filling and rinsing steps of the injection method is ≈135 sec. In Table 2 we present the properties of the injection method and in Fig. 2 we plot the water concentration and the *δ*D signal over a sequence of four injection pulses.

**Table 2:** PAL Autosampler Injection method.

Property	Value
Syringe Volume	10 *μ*l
Sample Volume	3.8 *μ*l
Fill Speed	400 nl/s
Fill Strokes	1
Pullup Delay	2.0 s
Inject Speed	50 nl/s
Pre Injection Delay	0 s
Post Injection Delay	10 s

#### Tray configuration - PAL Autosampler jobs sequence

In Table 3 we describe the sequence of the PAL autosampler jobs and the tray configuration. The measurement run begins with the VSMOW-SLAP calibration block (Vials #1 and #3) followed by the unknown (with the term “unknown” here we refer to the ice core samples whose isotopic composition is to be measured) samples run sequentially with increasing depth (Vials #4–56). A measurement of every local water standard consists of 20 injections of which the first 12 are discarded, in order to account for memory effects caused by the large isotopic step between the standards. The measurement of the unknowns is based on a block of four injections of which the first one is discarded. Three blocks containing a “check” standard that is treated as an unknown (Vial #2) are introduced in the middle and at the end of the VSMOW-SLAP calibration as well as at the end of the run after all unknowns have been measured. The “check” standard provides a measure of the obtained accuracy as well as the long term stability of the run. In total, 300 injections are performed, resulting to a total run duration of approximately 11 hours and 40 minutes. A complete run is illustrated in Fig. 2.

**Table 3:** Description of the sequence of PAL Autosampler jobs throughout a full measurement run.

Job	From Vial	To Vial	Inj per Vial	Discard	Description
1	1	3	20	12	VSMOW/SLAP calibration block
2	2	2	8	5	Standard 2 “check”
3	4	4	8	5	1st unknown
4	5	56	4	1	Unknowns 2–53
5	2	2	16	10	Standard 2 “check”

#### VSMOW-SLAP calibration protocols

The raw isotope measurements are calibrated on the VSMOW-SLAP scale using a two fixed-point calibration similar to^27^ and following the IAEA recommended procedures. For the record presented here, we have used 4 water isotopic standards calibrated against the primary IAEA reference waters VSMOW and SLAP (Table 4). The standards were combined in two triplets [−22, NEEM, −40] and [NEEM, −40, DC-02]. The first triplet [−22, NEEM, −40] is used for sections of the Holocene, whereas the more isotopically depleted samples during the Glacial are calibrated using the second triplet [NEEM, −40, DC-02].

**Table 4:** Copenhagen local standards used for the NEEM record. All values are given in ‰ with respect to VSMOW.

Name	*δ*^18^O	*δ*D
−22	−21.88	−168.3
NEEM	−33.5	−257.1
−40	−39.93	−310.7
DC-02	−54.07	−428.2

The two extreme standards of every triplet are used in order to define the slope and the intercept of the calibration line

1 $${\delta }_{{\rm{vsmow}}}=\alpha \,{\delta }_{{\rm{raw}}}+\beta $$

where the slope *α* and the intercept *β* are for the example of the low water standards triplet equal to:

2 $$\alpha =\frac{{\delta }_{{\rm{NEEMvsmow}}}-{\delta }_{{\rm{DC02Mvsmow}}}}{{\delta }_{{\rm{NEEMraw}}}-{\delta }_{{\rm{DC02raw}}}}$$

3 $$\beta ={\delta }_{{\rm{NEEMvsmow}}}-\frac{{\delta }_{{\rm{NEEMvsmow}}}-{\delta }_{{\rm{DC02vsmow}}}}{{\delta }_{{\rm{NEEMraw}}}-{\delta }_{{\rm{DC02raw}}}}\,{\delta }_{{\rm{NEEMraw}}}$$

From Eqs. 1–3 we calculate the VSMOW calibrated value of the middle “check” standard, which is then compared to the assigned VSMOW value of the “check” standard water in Table 4.

### Data analysis

#### Data analysis for individual runs

Upon completion, individual runs are processed using a spreadsheet-based data analysis scheme. The scheme performs the calculations of the VSMOW-SLAP calibration slope and intercept and subsequently applies them to the raw data, yielding a VSMOW-SLAP calibrated dataset. The data analysis sheet also calculates and reports the uncertainties of each measurement block (water standards and unknown samples). The main quality-control metric of the protocol is the calibrated value of the “check” standard, as well as the standard deviation calculated based on the three “check” standard blocks. The first value gives an estimate of the measurement’s accuracy, while the second is an estimate of the individual run’s noise level. The data analysis scheme selects the appropriate number of injections to be considered as described in Sec. 2 and generates a list of the analysed samples with their unique ID’s and isotopic composition expressed in per mille. Possible outliers in the run can occur in the rare case of faulty sample injections. These outliers are identified by visual inspection of the run results, as well as an inspection of the injection level and stability of the suspected unknowns. These data points are marked and then filled by means of linear interpolation. The record contains a total of 251 interpolated points accounting for 0.9% of the record (Table 1).

#### Post processing of the record

Compilation of the full record based on the individual run’s spreadsheets is performed using a Python routine that reads the data of interest sequentially. The Python routine reads the samples ID’s and their respective isotopic composition and assigns a section bottom depth to every sample. Additionally, metadata of interest are collected from each individual run and compiled on a separate file. These data concern sample injection characteristics, metrics related to the check standard as well as the values related to the VSMOW-SLAP calibration line.

#### The age scale

The GICC05 (Greenland Ice Core Chronology 2005)^35^, GICC05modelext^36^ and AICC2012 (Antarctic Ice Core Chronology 2012)^37,38^ timescales have all been transferred to NEEM high resolution depths using the approaches of^33,39,40^.

The GICC05 NEEM depths down to 1335.85 m have been transformed to NGRIP1 depths by linear interpolation between ECM match points and tephra horizons, and from these, GICC05 ages were obtained using the NGRIP1 GICC05 depth-age relationship. For the section from 1335.90 m to 1955.90 m, the NEEM depths have been transformed to NGRIP2 depths by linear interpolation between ECM match points and tephras, and from these, GICC05 ages were obtained using the NGRIP2 GICC05 depth-age relationship (see^39^ for matching details between NGRIP1, NGRIP2 and NEEM). Note that GICC05 comes with an estimate of the Maximum Counting Error (MCE) of the time scale^9^. In a standard deviation context, the maximum counting error should be regarded as 2-*σ*0^41^.

The GICC05modelext time scale has been transferred to NEEM depths in the following ways: Between 1955.95 m to 2203.55 m, the NEEM depths have been transformed to NGRIP2 depths by linear interpolation between ECM match points and tephras, and from these, GICC05modelext ages were obtained using the NGRIP2 GICC05modelext depth-age relationship (see^39^ for matching details). Below 2203.55 m NEEM depths, the ice in the NEEM core contains time reversals due to foldings. A detailed discussion of the matching and GICC05modelext timescale transfer between NEEM and NGRIP in this folded part of the core can be found in^33^. The depth-age relationship for this section is interpolated to 5 cm resolution from the lower resolution relationship given in the supplementary data file in^33^.

The AICC2012 time scale has been transferred to NEEM depths in the following ways: For ages younger than 60.2ka (1955.95 m NEEM depth) AICC12 is constructed to be practically identical to GICC05^38^, so no separate age transfer is needed. Between 1955.95 m to 2196.95 m, the NEEM depths have been transformed to NGRIP2 by linear interpolation between ECM match points and tephras, and from these, AICC2012 ages were obtained using the NGRIP2 AICC2012 depth-age relationship (see^39^ for matching details, and^38^ for the NGRIP2 AICC2012 timescale). For the section below 2196.95 m the ice in the NEEM core contains time reversals due to foldings. The depth-age relationship for this section is derived from a matching of the NEEM and EDML cores^33^. The lower resolution NEEM-EDML1 timescale given in the^33^ supplementary data has been interpolated to 5 cm resolution, and EDML1 ages have subsequently been converted to AICC2012 ages using the relationship between EDML1 and AICC2012 (see^38,42^ and^40^). It should be noted, that transferring AICC2012 to NEEM from NGRIP2 and from EDML does not yield the exact same ages for all NEEM depths due to matching uncertainties. Between 2196.95 m and 2197.00 m the two transfers of AICC2012 do, however, yield intersecting ages. Hence, to ensure continuity of the NEEM AICC2012 time scale, the AICC2012 ages are transferred from NGRIP2 down to 2196.95 m NEEM depth, while AICC2012 ages are transferred from EDML starting from 2197.00 m NEEM depth.

## Data Records

**Fig. 3:**
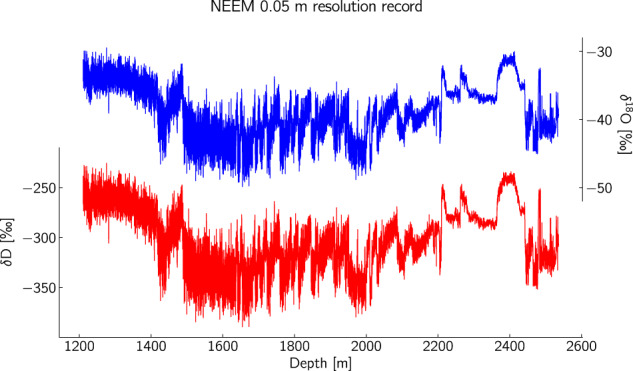
The compiled NEEM record from the depth of 1210.5 (corresponding to 8000 y b2k) and below.

**Fig. 4:**
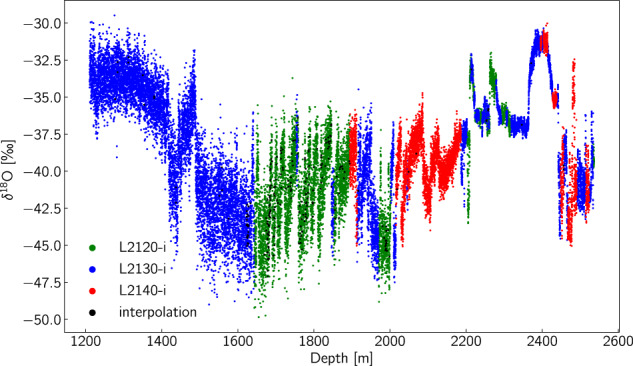
*δ*^18^O record and the coverage obtained for every instrument type.

The dataset^32^ can be found in the PANGAEA data repository (10.1594/PANGAEA.925552). In Table 5 we list the variables included in the data record together with a short description of each one of them. The full record is plotted in Fig. 3 (*δ*D and *δ*^18^O) whereas in Fig. 4 we present the contribution of every CRDS model to the measurement of the total record.

**Table 5:** Name and description of the variables included in the data file.

Column	Name	Unit	Description
1	Depth	m	Bottom Depth of each discrete sample
2	Age	y b2k	Age on the GICC05 counted time scale
3	Age	y b2k	Age on the GICC05 model extended time scale
4	Age	y b2k	Age on the AICC2012 time scale
5	MCE	y	Maximum Counting Error for the GICC05 time scale
6	*δ*^18^O	‰	*δ*^18^O of the sample
7	*δ*D	‰	*δ*D of the sample
8	*δ*^18^O std dev	‰	Run precision estimate for *δ*^18^O
9	*δ*D std dev	‰	Run precision estimate for *δ*D
10	Offset	‰	Run accuracy estimate for *δ*^18^O
11	Offset	‰	Run accuracy estimate for *δ*D

## Technical Validation

### Measurement precision and accuracy

The measurement precision of every run, is estimated from the calculation of the standard deviation of the valid injections of the “check” standard blocks (Vial #2). Measurement precision is given for *δ*^18^O and *δ*D in columns 8 and 9 of the data set. Similarly, the difference between the assigned and the post-calibration value of the “check” standard is used as a measure of the accuracy of the run. This metric is given in columns 10 and 11 for *δ*^18^O and *δ*D respectively.

**Fig. 5:**
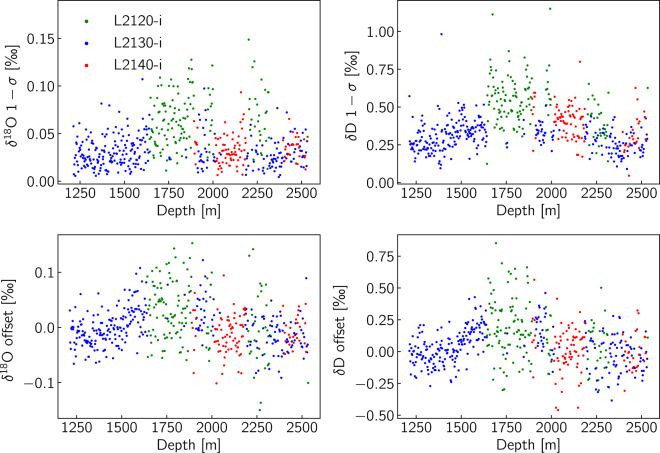
Quality-control metrics based on the measurement of the “check” standard. The top row presents the precision level of every measurement run while at the bottom row we present the offset of the “check” standard measurement from its assigned value- a measure of accuracy for every measurement run.

**Fig. 6:**
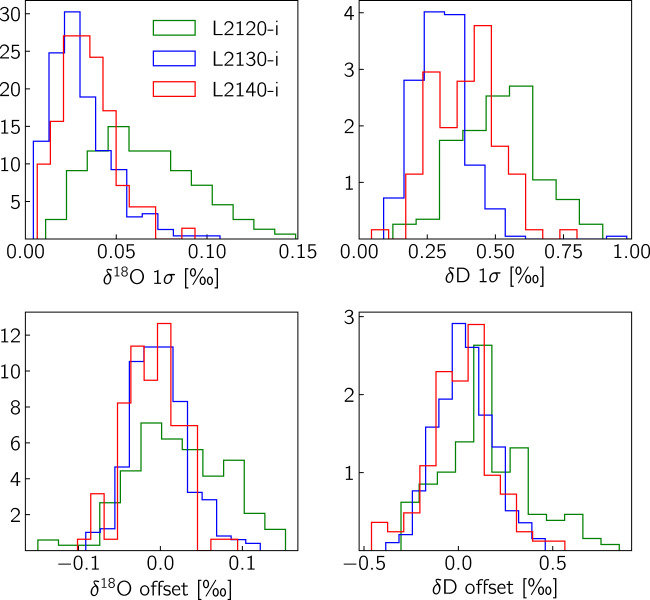
Histograms of the quality-control metrics of Fig. 5.

In Fig. 5 we plot the two metrics as a function of depth categorised by the instrument type and in Fig. 6 we bin the data points in histograms. From these two figures it is apparent that the performance of the L2130-i and L2140-i versions of the CRDS spectrometer is superior to that of the L2120-i version. The difference in performance concerns both the precision and the accuracy of the measurement and it appears to be more profound for the *δ*^18^O measurement. This behaviour is explained by the more accurate spectroscopic corrections, as well as the more precise control of the optical cavity’s temperature and pressure of the newer L2130-i and L2140-i models. The statistics of the distributions for the two metrics are outlined in Table 6. Based on the data of Table 6 we can commend that the obtained accuracy as expressed by the “check” standard offset, is well within the 2*σ* (95%) interval as defined by the precision of the “check” standard measurement for all the runs of the record.

**Table 6:** Statistics of the “check” standard quality-control metrics as presented in Fig. 6 for the three instrument types.

Instrument	*δ*^18^O precision [‰]	*δ*^18^O accuracy [‰]	*δ*D precision [‰]	*δ*D accuracy [‰]
L2120-i	0.065 ± 0.028	0.023 ± 0.059	0.524 ± 0.163	0.151 ± 0.232
L2130-i	0.031 ± 0.017	−0.001 ± 0.035	0.301 ± 0.098	0.034 ± 0.146
L2140-i	0.034 ± 0.015	−0.011 ± 0.033	0.390 ± 0.118	0.012 ± 0.172

## Data Availability

The Python code used for the transfer, organising of the data, estimation of the precision and accuracy metrics as well as the plots included in this manuscript can be found in https://github.com/vgkinis/neem_isotope_data_descriptor_code. In the repository, we also provide auxiliary code with basic routines for post-processing of the PANGAEA data file.

## References

[1] Dansgaard W (1964). Stable isotopes in precipitation. Tellus.

[2] Ciais P, Jouzel J (1994). Deuterium and oxygen-18 in precipitation - isotopic model, including mixed cloud processes. J. Geophys. Res.-Atmospheres.

[3] Jouzel J (1997). Validity of the temperature reconstruction from water isotopes in ice cores. J. Geophys. Res.-Oceans.

[4] Jouzel J (2013). A brief history of ice core science over the last 50 yr. Clim. Past.

[5] NGRIP members (2004). High-resolution record of Northern Hemisphere climate extending into the last interglacial period. Nature.

[6] EPICA Community Members (2004). Eight glacial cycles from an Antarctic ice core. Nature.

[7] Steffensen JP (2008). High-resolution Greenland ice core data show abrupt climate change happens in few years. Science.

[8] Vinther BM (2006). A synchronized dating of three Greenland ice cores throughout the Holocene. J. Geophys. Res.-Atmospheres.

[9] Rasmussen SO (2006). A new Greenland ice core chronology for the last glacial termination. J. Geophys. Res..

[10] Masson-Delmotte V (2005). GRIP Deuterium excess reveals rapid and orbital-scale changes in Greenland moisture origin. Science.

[11] Gkinis V, Simonsen SB, Buchardt SL, White JWC, Vinther BM (2014). Water isotope diffusion rates from the NorthGRIP ice core for the last 16,000 years - Glaciological and paleoclimatic implications. Earth and Planetary Science Letters.

[12] Buizert C (2014). Greenland temperature response to climate forcing during the last deglaciation. Science (New York, N.Y.).

[13] Jones TR (2018). Southern Hemisphere climate variability forced by Northern Hemisphere ice-sheet topography. Nature.

[14] Holme C, Gkinis V, Vinther BM (2018). Molecular diffusion of stable water isotopes in polar firn as a proxy for past temperatures. Geochim. Cosmochim. Acta.

[15] Bigeleisen J, Perlman ML, Prosser HC (1952). Conversion of Hydrogenic Materials to Hydrogen for Isotopic Analysis. Analytical Chemistry.

[16] Epstein T, Mayeda S (1953). Variations of ^18^O content of waters from natural sources. Geochim. Cosmochim. Acta.

[17] Vaughn BH (1998). An automated system for hydrogen isotope analysis of water. Chemical Geology.

[18] Begley IS, Scrimgeour CM (1997). High-precision *δ*^2^H and *δ*^18^O measurement for water and volatile organic compounds by Continuous-Flow Pyrolysis Isotope Ratio Mass Spectrometry. Analytical Chemistry.

[19] Gehre M, Geilmann H, Richter J, Werner RA, Brand WA (2004). Continuous flow ^2^H/^2^H and *δ*^18^O analysis of water samples with dual inlet precision. Rapid Communications In Mass Spectrometry.

[20] Kerstel ERT, van Trigt R, Dam N, Reuss J, Meijer HAJ (1999). Simultaneous determination of the ^2^H/^1^H, ^17^O/^16^O and ^18^O/^16^O isotope abundance ratios in water by means of laser spectrometry. Analytical Chemistry.

[21] Van Trigt R, Meijer HAJ, Sveinbjörnsdóttir AE, Johnsen SJ, Kerstel ERT (2002). Measuring stable isotopes of Hydrogen and Oxygen in ice by means of laser spectrometry: the Bølling transition in the Dye-3 (south Greenland) ice core. Ann. Glaciol..

[22] Crosson ER (2008). A cavity ring-down analyzer for measuring atmospheric levels of methane, carbon dioxide, and water vapor. Applied Physics B-Lasers And Optics.

[23] Gkinis V, Popp TJ, Johnsen SJ, Blunier T (2010). A continuous stream flash evaporator for the calibration of an IR cavity ring-down spectrometer for the isotopic analysis of water. Isotopes In Environmental and Health Studies.

[24] Aemisegger F (2012). Measuring variations of *δ*^18^O and *δ*^2^H in atmospheric water vapour using two commercial laser-based spectrometers: an instrument characterisation study. Atmos. Meas. Tech..

[25] Steen-Larsen HC (2013). Continuous monitoring of summer surface water vapor isotopic composition above the Greenland Ice Sheet. Atmos. Chem. Phys..

[26] Benetti, M. *et al*. Stable isotopes in the atmospheric marine boundary layer water vapour over the Atlantic Ocean, 2012–2015. *Sci. Data* 160128, 10.1038/sdata.2016.128 (2017). 10.1038/sdata.2016.128 PMC5240618 28094798

[27] Gkinis V (2011). Water isotopic ratios from a continuously melted ice core sample. Atmos. Meas. Tech..

[28] Maselli, O. J., Fritzsche, D., Layman, L., McConnell, J. R. & Meyer, H. Comparison of water isotope-ratio determinations using two cavity ring-down instruments and classical mass spectrometry in continuous ice-core analysis. *Isotopes Environ. Health Stud*. 387–398, 10.1080/10256016.2013.781598 (2013). 10.1080/10256016.2013.781598 23713832

[29] Emanuelsson BD, Baisden WT, Bertler NAN, Keller ED, Gkinis V (2015). High-resolution continuous-flow analysis setup for water isotopic measurement from ice cores using laser spectroscopy. Atmos. Meas. Tech..

[30] Jones TR (2017). Improved methodologies for continuous-flow analysis of stable water isotopes in ice cores. Atmos. Meas. Tech..

[31] Jones TR (2017). Water isotope diffusion in the WAIS Divide ice core during the Holocene and last glacial. J. Geophys. Res. Earth Surf..

[32] Gkinis V (2020).

[33] NEEM Community Members (2013). Eemian interglacial reconstructed from a Greenland folded ice core. Nature.

[34] Rasmussen SO (2014). A stratigraphic framework for abrupt climatic changes during the Last Glacial period based on three synchronized Greenland ice-core records: refining and extending the INTIMATE event stratigraphy. Quat. Sci. Rev..

[35] Svensson A (2008). A 60 000 year Greenland stratigraphic ice core chronology. Clim. Past.

[36] Wolff EW, Chappellaz J, Blunier T, Rasmussen SO, Svensson A (2010). Millennial-scale variability during the last glacial: The ice core record. Quat. Sci. Rev..

[37] Bazin L (2013). An optimized multi-proxy, multi-site Antarctic ice and gas orbital chronology (AICC2012): 120-800 ka. Clim. Past.

[38] Veres D (2013). The Antarctic ice core chronology (AICC2012): an optimized multi-parameter and multi-site dating approach for the last 120 thousand years. Clim. Past.

[39] Rasmussen SO (2013). A first chronology for the North Greenland Eemian Ice Drilling (NEEM) ice core. Clim. Past.

[40] Govin A (2015). Sequence of events from the onset to the demise of the Last Interglacial: Evaluating strengths and limitations of chronologies usedÂ in climatic archives. Quat. Sci. Rev..

[41] Andersen KK (2006). The Greenland Ice Core Chronology 2005, 15-42 ka. Part 1: constructing the time scale. Quat. Sci. Rev..

[42] Ruth U (2007). “EDML1”: a chronology for the EPICA deep ice core from Dronning Maud Land, Antarctica, over the last 150 000 years. Clim. Past.

